# Towards Cost-Effective Operational Monitoring Systems for Complex Waters: Analyzing Small-Scale Coastal Processes with Optical Transmissometry

**DOI:** 10.1371/journal.pone.0170706

**Published:** 2017-01-20

**Authors:** Marta Ramírez-Pérez, Rafael Gonçalves-Araujo, Sonja Wiegmann, Elena Torrecilla, Raul Bardaji, Rüdiger Röttgers, Astrid Bracher, Jaume Piera

**Affiliations:** 1 Department of Physical and Technological Oceanography, Institute of Marine Sciences (ICM-CSIC), Barcelona, Spain; 2 Phytooptics Group, Physical Oceanography of Polar Seas, Climate Sciences Division, Alfred Wegener Institute Helmholtz Centre for Polar and Marine Research, Bremerhaven, Germany; 3 Remote Sensing Department, Institute for Coastal Research, Centre for Materials and Coastal Research, Helmholtz-Zentrum Geesthacht, Geesthacht, Germany; 4 Institute of Environmental Physics, University of Bremen, Bremen, Germany; CNRS, FRANCE

## Abstract

The detection and prediction of changes in coastal ecosystems require a better understanding of the complex physical, chemical and biological interactions, which involves that observations should be performed continuously. For this reason, there is an increasing demand for small, simple and cost-effective in situ sensors to analyze complex coastal waters at a broad range of scales. In this context, this study seeks to explore the potential of beam attenuation spectra, c(λ), measured in situ with an advanced-technology optical transmissometer, for assessing temporal and spatial patterns in the complex estuarine waters of Alfacs Bay (NW Mediterranean) as a test site. In particular, the information contained in the spectral beam attenuation coefficient was assessed and linked with different biogeochemical variables. The attenuation at λ = 710 nm was used as a proxy for particle concentration, TSM, whereas a novel parameter was adopted as an optical indicator for chlorophyll a (Chl-a) concentration, based on the local maximum of c(λ) observed at the long-wavelength side of the red band Chl-a absorption peak. In addition, since coloured dissolved organic matter (CDOM) has an important influence on the beam attenuation spectral shape and complementary measurements of particle size distribution were available, the beam attenuation spectral slope was used to analyze the CDOM content. Results were successfully compared with optical and biogeochemical variables from laboratory analysis of collocated water samples, and statistically significant correlations were found between the attenuation proxies and the biogeochemical variables TSM, Chl-a and CDOM. This outcome depicted the potential of high-frequency beam attenuation measurements as a simple, continuous and cost-effective approach for rapid detection of changes and patterns in biogeochemical properties in complex coastal environments.

## Introduction

Coastal regions are highly dynamic and productive ecosystems, with high ecological and economic values [1]. They are also vulnerable areas subjected to considerable anthropogenic pressures through urban and industrial development, pollution, fisheries, agriculture and aquaculture, recreation, etc. These pressures have caused, in many cases, habitat degradation carrying serious environmental and economic consequences, such as harmful algal blooms (HABs), anoxia, accumulation of pollutants and toxins or over-exploited fisheries [2]. Added to this, the effects of climate change and natural hazards are threatening the capability of coastal ecosystems to support goods and valuable services [3]. For these reasons, increasing national and international efforts have been addressed over the last decades to establish and implement environmental strategies for preservation, conservation and sustainable use of these ecosystems. In accordance with the requirements of the European Water Framework Directive (WFD, 2000/60/EC) and the Marine Strategy Framework Directive, new interdisciplinary research programs are successfully being carried out such as the coastal module of the Global Ocean Observing System (Coastal GOOS), the Coastal Observing System for Northern and Arctic Seas (COSYNA), the Global Earth Observations System of Systems (GEOSS) or the Marine Geological and Biological Habitat Mapping (GEOHAB). All those programs have been conceived to monitor, forecast and assess the state of coastal ecosystems, which involve integrated, multidisciplinary and multiscale observing systems. Coastal environments are governed by complex physical and biogeochemical processes and thus, undergo changes over a broad range of time-space scales. Continuous and routine provision of data is therefore required to assess the states of these ecosystems, detect changes in these states and evaluate their impacts [4]. In addition, there is a demand for compact, inexpensive, stable and low power in situ sensors to enable sustainable and long-term monitoring.

A powerful solution to resolve in-water variability at a wide range of space and time scales is the use of in situ measurements of Inherent Optical Properties (IOPs) [5–8]. IOPs depend on the composition, morphology, and concentration of the particulate and dissolved chromophoric substances in the water and thus, can be used to estimate some water quality variables (e.g. turbidity) [9] and biogeochemical properties (e.g. Chlorophyll a and suspended matter concentrations) [10–11]. Among the IOP measuring devices, transmissometers present numerous advantages due to their general availability and simplicity of both operation and data processing [12]. These devices have been used to estimate the concentration of the suspended material in water [13], the composition [11] and size distribution of particles [14] and the particulate organic carbon [8, 15]. Furthermore, several studies have demonstrated the relationships between particulate attenuation, c_p_, and chlorophyll concentration (as an index of phytoplankton biomass) in oceanic waters [16–18]. In coastal waters, c_p_ also registers changes in inorganic, detrital, and heterotrophic particles, thus compromising its correlation with chlorophyll a (Chl-a) concentration [18]. Nevertheless, the correlation between both variables (i.e. c_p_ and Chl-a) still needs to be further explored.

Recent technological advances have led to the development of high spectral resolution (i.e. hyperspectral), cost-effective, compact and low power transmissometers (e.g. VIPER, TriOS GmbH [19]), which have improved the operational capabilities. In this context, this study evaluates the potential of economically affordable and advanced-technology transmissometers to detect changes and patterns in the biogeochemical properties at high temporal and spatial resolution in complex coastal environments. In particular, we focus on the information contained in the spectral beam attenuation coefficient and explore its suitability as a qualitative proxy for different biogeochemical properties. The observed patterns are analyzed in relation to the prevailing physical forcing to better understand the biophysical coupling.

This study is focused on the microtidal estuary of Alfacs Bay (Ebro Delta, NW Mediterranean coast), using it as a test site. This bay is an important shellfish production area commonly affected by HABs events, which lead to significant economic losses [20]. For this reason, this area has been intensively monitored since 1990. Research efforts have focused on characterizing the hydrodynamics of this bay [21–25], its ecology [26–29] and the coupling between physical and biological processes [30]. However, the use of optical-based approaches in this area -which allow the assessment of fine-scaled temporal and spatial variability of water constituent characteristics- is still very limited. Only Busch (2013) [31] analyzed the phytoplankton dynamics in this environment using radiometric measurements, which provided useful data only in day time. One of the main conclusions of this author was that continuous observations in Alfacs Bay are required to properly understand the rapid ecosystem dynamics.

## Materials and Methods

### Study site

**Fig 1:**
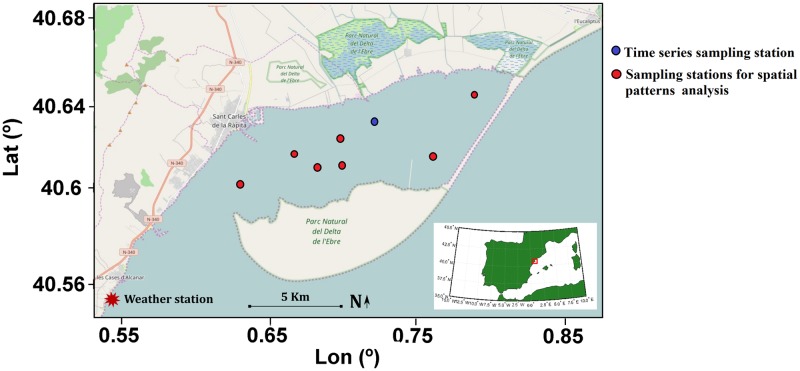
Location map of Alfacs Bay in NW Mediterranean Sea. The red star indicates the location of the weather station, whereas circles show the sampling stations for the analysis of temporal (blue circle) and spatial (red circles) patterns. Map produced with Open Street Map.

Alfacs Bay is located in the south of the Ebro River Delta (Spain), in the NW Mediterranean Sea (Fig 1). It is a shallow estuarine bay with 11 km length, 4 km width and a maximum depth of 6.5 m. It is a semi-enclosed embayment separated from the open sea by a sand barrier that leaves an opening of roughly 2.5 km width, allowing the exchange of water with the open sea. The major physical forcings in the bay are wind and freshwater input, whereas tidal forcing is negligible with a maximum range of 0.25 m [32]. The freshwater discharge is derived mainly from the rice-fields irrigation channels, located in the northern part of the Bay. These channels are open from April to October or November, with an average flux rate of ca. 14.5 m^3^·s^−1^ [22]. The freshwater inputs induce vertical stratification, while only during strong wind events the water column is vertically mixed [21]. Heat fluxes in the ocean-atmosphere boundary layer in summer periods contribute in addition to stratifying the water column [33].

### Field campaign

No specific permissions were required for the location of the field campaign, and the study did not involve endangered nor protected species. Two sampling strategies were adopted to analyze both temporal and spatial patterns in Alfacs Bay in June 2013. The analysis of temporal patterns was conducted from the 24^th^ of June at 9:30 pm for 48 hours. This time series of vertical profiles was gathered from 0.5 m to 3 m depth at a fixed station located in the north-central part of the Bay (blue circle in Fig 1). At this station, simultaneous measurements of physical (wind and current velocities and direction, and water temperature) and optical parameters (beam attenuation and near-forward angular scattering) were conducted continuously with a vertical resolution of 0.5 m. At each depth, instruments measured for 10 minutes. Thereby, each vertical profile took approximately 1 hour. Water samples were collected every 6 hours at three different depths (0.5 m, 1.5 m and 3 m) for later laboratory analysis of biogeochemical and optical parameters [Chl-a, total suspended matter (TSM), algal and non-algal particulate absorption (a_ph_ and a_nap_, respectively) and coloured dissolved organic matter (CDOM) absorption]. The analysis of the spatial variability was undertaken on the 27^th^ of June at seven stations along the bay (red circles in Fig 1). At each station, measurements of physical (temperature and salinity) and optical parameters were made with a ship deployed profiling package and water samples were collected at three different depths (0.5 m, 3 m and 0.5 m above the bottom). Instruments measured for 5 minutes every 0.5 m along each depth profile.

#### Physical parameters

Wind data were obtained from the weather station nearby the coastline in Les Cases de Alcanar, ca. 5 km south of Alfacs Bay (Fig 1). Three-dimensional current velocities were measured with an upward-looking Acoustic Doppler Current Profiler (ADCP, 2MHz Aquadopp, Nortek) moored at roughly 2 m depth since the maximum depth at this station was 3.5 m. It was configured to record 10-min average data with vertical cells of about 25 cm.

Water temperature and salinity were measured with the CTD48M (Sea&Sun Technology, Germany). Unfortunately, a failure in the instrument caused the loss of the data corresponding to the temporal analysis at the fixed sampling station. Temperature data provided by the multiple-parameter system LISST (Sequoia Scientific Inc.) were used instead.

#### Optical measurements

Spectral beam attenuation coefficient was measured with the 25-cm path length VIPER (TriOS GmbH., Germany [19]). It is an open-path hyperspectral transmissometer which measures the beam attenuation, c(λ), in the spectral range from 360 nm to 750 nm, with an optical resolution of 15 nm (defined by the FWHM) and an acceptance angle of 0.8°. More detailed information about the instrument performance and validation can be found in Ramírez-Pérez *et al*. (2015) [34]. VIPER measurements were carefully performed (i.e. on the shadow side of the ship and under calm sea surface conditions) to avoid ambient light contamination [34]. c(λ) data were collected continuously and averaged over 5 minutes. Milli-Q water references were subtracted and data were corrected for temperature and salinity dependence of pure seawater to derive the total non-water beam attenuation spectrum, c_pg_(λ) [34]. Measurements of particle size distribution (PSD) from 1.25 μm to 250 μm were conducted with the LISST-100X (Sequoia Scientific, Inc.). This instrument measures the near-forward scattering at 32 logarithmically spaced angles and the beam attenuation at 670 nm [35]. A successfully performance analysis between the attenuation measured at 670 nm by LISST and VIPER was previously carried out [34]. Therefore, this study focused only on the LISST scattering data to derive the particle size distribution. The volume concentration, *V(*D*)*, was obtained through inversion of the angular forward scattering pattern using the manufacturer-provided inversion routine. The used inversion algorithm is based on a kernel matrix derived from Mie theory of scattering by spherical particles. Data from the outer and inner rings were excluded from further analysis due to the instability observed in the smallest and largest size ranges [36]. Then, the particle number distribution, *N*(D), was calculated from the equation:

$$N\left(D\right)=6\cdot V\left(D\right)/\left(\pi D^{3}\right)$$ (1)

where D represents the diameter of a volume-equivalent sphere for the midpoint of each size class. To obtain the PSD, the average number of particles in each size class was divided by the width of the class, which is denoted as *N'*(D). Finally, the PSD was fitted to the power-law function (or Junge distribution) [37]:

$$N^{\prime }\left(D\right)=N^{\prime }\left(D_{0}\right){\left(\frac{D}{D_{0}}\right)}^{-\xi }$$ (2)

where D_0_ is a reference diameter, *N'*(D_0_) the differential number concentration at D_0_ and ξ is the nondimensional PSD slope.

#### Laboratory analysis of water samples

- CDOM absorption measurements: absorbance spectra (240–600 nm) were acquired with the Aqualog fluorescence spectrometer (HORIBA JobinYvon, Germany) directly after sampling. Water samples were syringe-filtered with 0.2 μm Whatman Spartan filters before analysis with 1 cm quartz cuvettes. Absorbance measurements were further converted to absorption coefficient, which is used as a proxy for the CDOM content in a given water sample. The Napierian absorption coefficient of CDOM at each wavelength (a_λ_) was obtained from the given equation:
  $$a_{\lambda }\left(m^{-1}\right)=\left(2.303\cdot A_{\lambda }\right)/L$$ (3)
  where A_λ_ is the absorbance at specific wavelength and *L* is the cuvette path length in meters. More detailed information about the measurement protocol can be found in Gonçalves-Araujo *et al*. (2015) [38]. CDOM absorption spectra, a(λ), were fitted to the following exponential function [39]:
  $$a\left(\lambda \right)=a\left(\lambda_{0}\right)\cdot e^{-S\left(\lambda -\lambda_{0}\right)}$$ (4)
  where S represents the spectral slope and a(λ_0_), the absorption coefficient at a reference wavelength λ_0_ (443 nm in this case). The function was fitted to the wavelength range from 300 to 600 nm and extrapolated to 720 nm for later analysis of CDOM contribution at longer wavelengths.
- Algal and non-algal particulate absorption (a_ph_(λ) and a_nap_(λ)): water samples were immediately filtered through ø 47-mm GF/F filters (pore size 0.7 μm), shock-frozen in liquid nitrogen and stored at -80°C until analysis in the laboratory at the Alfred-Wegener-Institute. The partition of the particulate absorption, a_p_(λ), into phytoplankton, a_ph_(λ), and non-algal absorption, a_nap_(λ), was performed by the filter pad technique following Ferrari and Tassan (1999) [40]. We used a Cary 4000 UV/VIS dual beam spectrophotometer equipped with a 150-mm integrating sphere (Varian Inc., USA) as described in Taylor *et al*. (2011) [41]. The measurement procedures and data analysis were performed as detailed in Röttgers and Gehnke (2012) [42]. Phytoplankton absorption a_ph_ was obtained as the difference between a_p_ and a_nap_.
- Chlorophyll a: water samples for phytoplankton pigments analysis were filtered immediately after collection through ø 25-mm Whatman GF/F filters (pore size 0.7 μm). Then, filters were shock-frozen in liquid nitrogen and stored at -80°C until analysis in the laboratory at the Alfred-Wegener-Institute. The extracted pigments were analyzed using the High Performance Liquid Chromatography (HPLC) technique following the method of Barlow *et al*. (1997) [43], with modification customized to our instruments as detailed in Taylor *et al*. (2011) [41]. In this study, we use the Chl-a concentration as an index of phytoplankton biomass and covarying materials (biogenic detritus).
- Total suspended matter concentration: TSM concentration was determined gravimetrically following Röttgers *et al*. (2014) [44] to reject potential errors associated with salt retention in the filters and loss of materials during washing and combustion [44–45]. Thereby, four different volumes of each water sample (within the range from 0.6 to 2.2 liters) were filtered immediately after collection through pre-weighed Whatman GF/F filters (ø 47 mm). Afterwards, the gained mass of each filter was determined by subtracting the weight of the filter from the final weight. A linear regression analysis was performed for filtered volume versus the gained mass, and the regression slope was taken as the TSM concentration value [44].

### Data and statistical analysis

**Fig 2:**
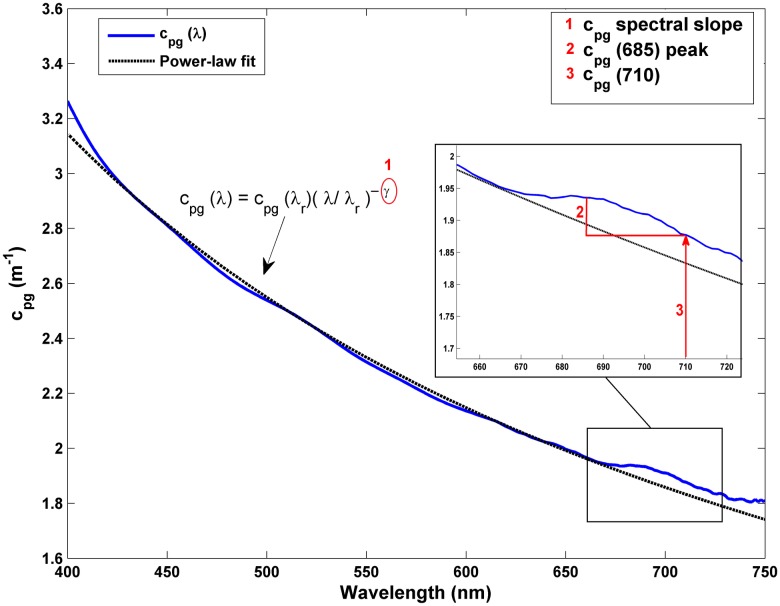
Representative total non-water beam attenuation spectrum measured in Alfacs Bay in June 2013. Numbers 1–3 indicate the three spectral features used in this study as proxies for biogeochemical variables: 1 = spectral slope of c_pg_(λ) for CDOM absorption, 2 = c_pg_(685)-c_pg_(710) for Chl-a and 3 = c_pg_(710) for TSM concentration.

This study explored the information contained in the beam attenuation spectrum as a proxy for different biogeochemical properties. In particular, the analysis focused on three major spectral features, which are described as follow (Fig 2):

- Spectral slope: it is the major spectral shape feature of the beam attenuation coefficient and is related to the particle size distribution and CDOM content [46]. For this reason and due to the lack of in situ CDOM absorption measurements, we used the total non-water beam attenuation spectral slope to detect variations in CDOM. This simplification was adopted because of the high CDOM content in Alfacs Bay [31] and the availability of additional particle size distribution measurements. To compute this parameter, beam attenuation spectra were fitted to the power-law function [46]:
  $$c_{pg}\left(\lambda \right)=c_{pg}\left(\lambda_{r}\right)\cdot {\left(\lambda /\lambda_{r}\right)}^{-\gamma }$$ (5)
  where λ_r_ is a reference wavelength (532 nm, in our case) and γ is the power-law slope. The exponent was derived by non-linear least-squares regression, with a r^2^>0.98 in all cases.
- Peak height associated with red band phytoplankton absorption peak: although c(λ) is typically a smoothly varying function of wavelength [46–47], deviations from its theoretical behavior -associated with absorbing particles- have been reported by several authors [48–50]. Zaneveld and Kitchen (1995) [48] observed step increases at the long-wavelength side of the chlorophyll absorption peaks as result of anomalous diffraction and dispersion [51], which was called "absorption peak effects". This local maximum is therefore expected to be related to the Chl-a content (in addition to other factors such as the particle size distribution) [48]. For this reason, the link between the local maximum of c(λ) and the Chl-a concentration was tested in this study, since it can provide a first estimate of phytoplankton biomass and covarying materials. Similarly to Davis *et al*. (1997) [52], who estimated the Chl-a concentration based on the red band absorption peak -a(676)- by subtracting a baseline, we computed the peak height in the red band of the beam attenuation spectrum. However, here the local maximum was found at 685 nm (approximately 10 nm past the absorption peak, in agreement with Zaneveld and Kitchen (1995) [48]). The attenuation at 710 nm was then subtracted from this local peak as a base value to remove the effect of particle scattering. This wavelength was empirically chosen based on providing the best results (based on r^2^ and RMSE as compared to collocated Chl-a data determined by HPLC at discrete samples). Thereby, the peak height was computed as c_pg_(685)-c_pg_(710), which was used as a proxy for Chl-a concentration.
- c_pg_(710): At long visible wavelengths, the attenuation is assumed to be determined mostly by their scattering properties and secondarily by the particulate absorption, whereas CDOM absorption has a insignificant contribution [15, 53–55]. For this reason, the attenuation in the red part of the visible spectrum (i.e. 660 and 670 nm) has been commonly used as proxy for suspended particle concentration, since it responds primarily to concentration and secondarily to size and nature of the particles [56]. While this assumption can be considered true in open waters, it could fail for complex coastal waters with high CDOM contents, which can yield a non-negligible CDOM absorption at long wavelengths (~700 nm) (e.g. [57]). Nevertheless, the exponential decrease of CDOM absorption with wavelength involves that the longer the wavelength, the smaller its contribution to the beam attenuation signal. For this reason, this study used the beam attenuation in the NIR (concretely at 710 nm) as proxy for TSM, where the CDOM absorption influence was minimum.

Variations in time and space of these optical parameters were analyzed by means of statistical techniques. In particular, the Kruskal-Wallis H-test was applied at 5% significance level (α = 0.05) in order to identify temporal and spatial patterns in Alfacs Bay, given that data were not normally distributed, as demonstrated by the Shapiro-Wilk test performed prior to analysis. On the other hand, since both inputs were subject to errors, a model II linear regression analysis was applied to investigate the relationships between optical parameters and biogeochemical variables. Additionally, the correlations between them were examined using non-parametric Spearman-r correlation coefficients and the associated errors were determined by means of the root mean squared error (RMSE).

## Results and Discussion

At first, the results from validating the above-mentioned beam attenuation-based proxies with laboratory-measured biogeochemical variables are presented. Secondly, the temporal and spatial variability and patterns of these optical and biogeochemical parameters in Alfacs Bay are shown.

### Validation of biogeochemical proxies

#### Attenuation at 710 nm *vs*. total suspended matter concentration

**Fig 3:**
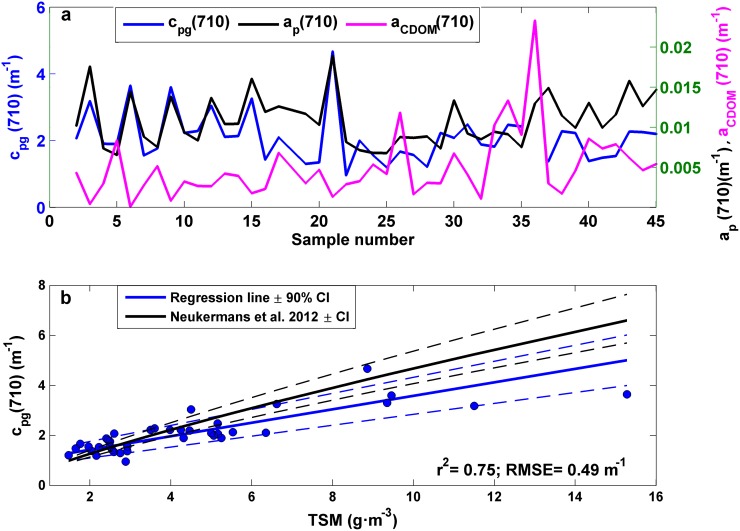
(a) Comparison of c_pg_(710), a_p_(710) and a_CDOM_ (710) along all the samples used in this study. (b) Scatter plot of TSM and the attenuation at 710 nm. Best fit ± 90% confidence intervals are shown in blue. Black solid and dashed lines represent the 90% prediction bounds of the [TSM]-c_p_(670 nm) data of Neukermans *et al*. (2012) (for the C-Star).

The comparison between the total non-water beam attenuation coefficient and the particulate and CDOM absorption at 710 nm (c_pg_(710), a_p_(710) and a_CDOM_(710), respectively) was performed to determine the relative contribution of the two last components to the bulk c_pg_(710) signal (Fig 3a). In all samples, a_p_(710) and a_CDOM_(710) represented a minor fraction of c_pg_(710), since their values were two orders of magnitude lower than c_pg_(710). c_pg_(710) oscillated from 0.96 to 4.66 m^-1^, whereas a_p_(710) and a_CDOM_(710) ranged from 0.0065 to 0.025 m^-1^ in our dataset. The insignificant CDOM contribution to the attenuation signal at 710 nm, enabled to use c_pg_(710) as proxy for TSM. Then, a model II linear regression analysis was applied to investigate the relationship between c_pg_(710) and TSM (Fig 3b). The regression slope (± SD) was 0.224±0.03 g·m^-2^, which agreed with previous works [53, 54]. In addition, although our slope was flatter, our observations were within the confidence bounds of the relationship found by Neukermans *et al*. (2012) [55] for the C-Star attenuation meter (with an acceptance angle of 1.2°) (Fig 3b). This disparity in the regression slope can be partly explained due to the different attenuation wavelength used in the relationship (670 and 710 nm in Neukermans *et al*. (2012) [55] and in our study, respectively). A significant correlation was observed between c_pg_(710) and TSM, with r^2^ = 0.75 and RMSE = 0.49 m^-1^ (p<0.001).

#### c_pg_(685) peak height *vs*. Chl-a concentration

**Fig 4:**
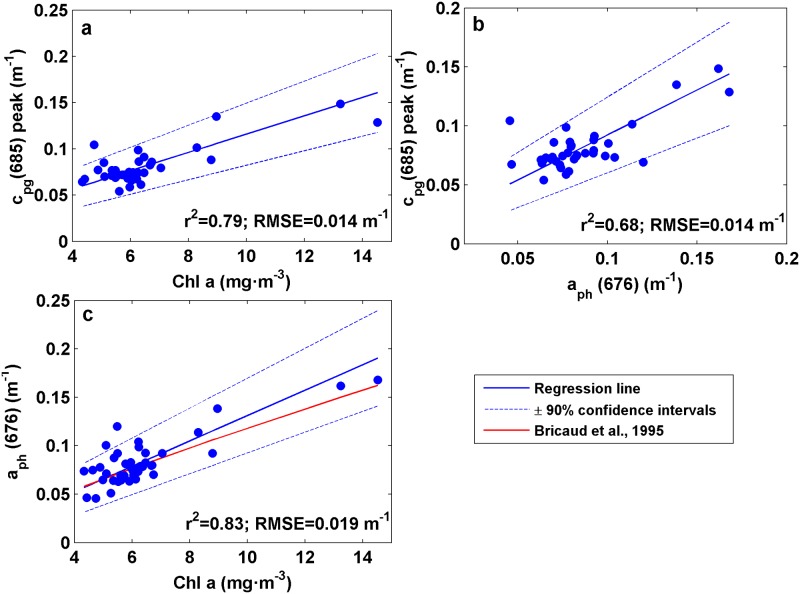
Scatter plots of (a) Chl-a concentration and c_pg_(685) peak. (b) Phytoplankton absorption at 676 nm, a_ph_(676), and c_pg_(685) peak. (c) Chl-a concentration and a_ph_(676). The red line represents the power-law fit proposed by Bricaud *et al*. (1995). Blue lines are the regression line ± 90% confidence intervals.

A relatively good linear correlation was found between the Chl-a concentration and the non-water attenuation peak height at 685 nm, c_pg_(685) peak (r^2^ = 0.79, RMSE = 0.014 m^-1^, p<0.001, Fig 4a). Thereby, it is reasonable to consider c_pg_(685) peak as a proxy for tracking changes in Chl-a concentration. Since this peak was associated with the red-band Chl-a absorption peak due to anomalous dispersion, the linear correlation between c_pg_(685) peak and the laboratory-derived phytoplankton absorption at 676 nm, a_ph_(676), was examined (Fig 4b). A significant linear correlation was also obtained in this case, with r^2^ = 0.68 and RMSE = 0.014 m^-1^ (p<0.01). In turn, the correlation between Chl-a concentration and a_ph_(676) was analyzed (r^2^ = 0.83; RMSE = 0.019 m^-1^; p<0.001) and compared to the power-law fit obtained by Bricaud *et al*. (1995) [58]. Our observations were in agreement with the function predicted by those authors (Fig 4c). According to Bricaud *et al*. (1995) [58], the relationship between a_ph_(λ) and Chl-a varied as a result of changes in packaging effect and pigment composition. Our proxy is therefore suspected to be affected not only by these factors but also by minor contributions associated with CDOM and non-algal particles absorption, particle size distribution or Chl-a fluorescence, which compromise the relationship found between Chl-a concentration and c_pg_(685) peak height. For this reason, we recommend this approach as a qualitative proxy, since its capability to provide quantitative estimates of Chl-a concentration should be further explored with a more extensive dataset. The potential influence of Chl-a fluorescence, which could lead to a decrease in the attenuation signal around 685 nm, was not evaluated here. Nevertheless, we assumed a minor effect since Chl-a fluorescence from the light beam leads to an emission into all directions, and therefore the amount of fluorescence into the direction of the beam towards the detector can be considered insignificant.

#### Spectral slope of total non-water beam attenuation *vs*. a_CDOM_(443)

**Fig 5:**
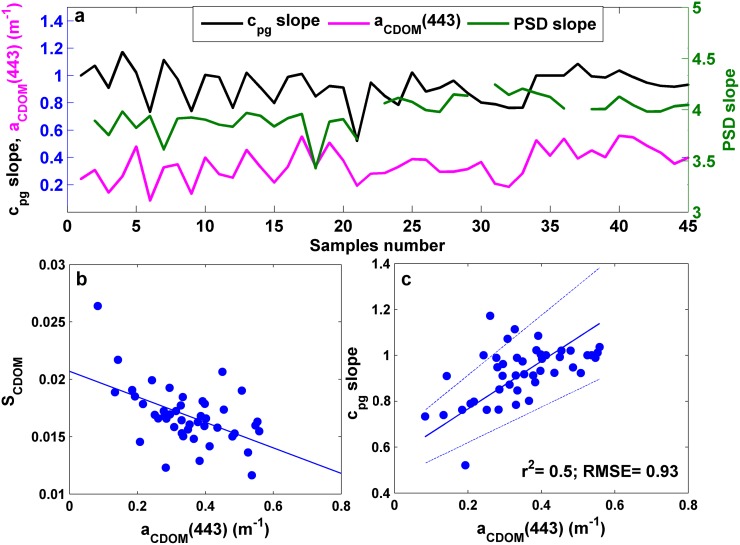
(a) Evolution of beam attenuation spectral slope (c_pg_ slope), PSD slope and CDOM absorption at 443 nm (a_CDOM_(443)) along all samples. (b) Scatter plot of a_CDOM_(443) and CDOM absorption spectral slope, S_CDOM_. (c) Scatter plot of c_pg_ slope and a_CDOM_(443). The blue lines are the regression fits.

The evolution of the attenuation spectral slope, the particle size distribution slope and the CDOM absorption at 443 nm was analyzed to evaluate the suitability of using c_pg_ slope as an indicator of CDOM content. Variations in c_pg_ slope responded mainly to changes in a_CDOM_(443), since both parameters exhibited a fairly similar behavior although the magnitude of these variations differed. PSD slope, however, varied within a relatively narrow range (from 3.43 to 4.24), playing a smaller role in the variations observed in c_pg_ slope (Fig 5a). Note that the PSD slope of the LISST and the VIPER-derived c_pg_ slope are sensitive to different particle range given the distinct scattering angles they collect, which can contribute to the different behavior observed among both variables. In order to test whether these variations in c_pg_ slope were associated with changes in the magnitude of a_CDOM_ instead of in the CDOM absorption spectral slope, S_CDOM_, the correlation between a_CDOM_(443) and S_CDOM_ was analyzed (p<0.001). An inverse relationship was found between both variables, which is consistent with the observations from Helms *et al*. (2008) [59] (Fig 5b). In contrast, no correlation was observed between S_CDOM_ and c_pg_ slope (p>0.1). Finally, the relationship between a_CDOM_(443) and c_pg_ slope showed a significant correlation (p<0.001), although the coefficient of determination was not too strong (r^2^ = 0.5; RMSE = 0.93). This correlation was due to the high CDOM content in Alfacs Bay [31]. For future studies, however, it is recommended to perform in situ measurements of 0.2 μm-filtered and unfiltered seawater alternatively to determine CDOM absorption separately (e.g. [14]).

### Spatial variability

**Fig 6:**
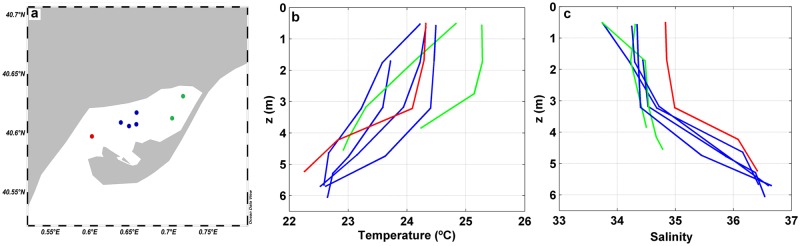
Analysis of the spatial variability in Alfacs Bay. (a) Sampling stations for the analysis of spatial patterns, with different colors according to its location: inner (green), central (blue), outer (red) region of the Bay. Map produced with Ocean Data View (ODV) software [60]. (b) Temperature and (c) salinity vertical profiles measured at the seven sampling stations with colors according to Fig 6a.

The horizontal and vertical spatial variability of the environmental, optical and biogeochemical parameters was analyzed based on vertical profiles measured at seven stations spread in Alfacs Bay (Fig 6a). The vertical profiles of temperature and salinity showed a stratified water column, with a fresher and warmer surface layer and an underlying cooler and saltier water layer (Fig 6b and 6c). The pycnocline was located at ~3.5m depth, consistent with previous studies [22, 32–33]. The averaged temperature and salinity gradients between surface and bottom were of ΔT = 1.33°C and ΔS = 1.84, with maximal differences of 2.1°C and 2.7, respectively (found in the bay mouth).

**Fig 7:**
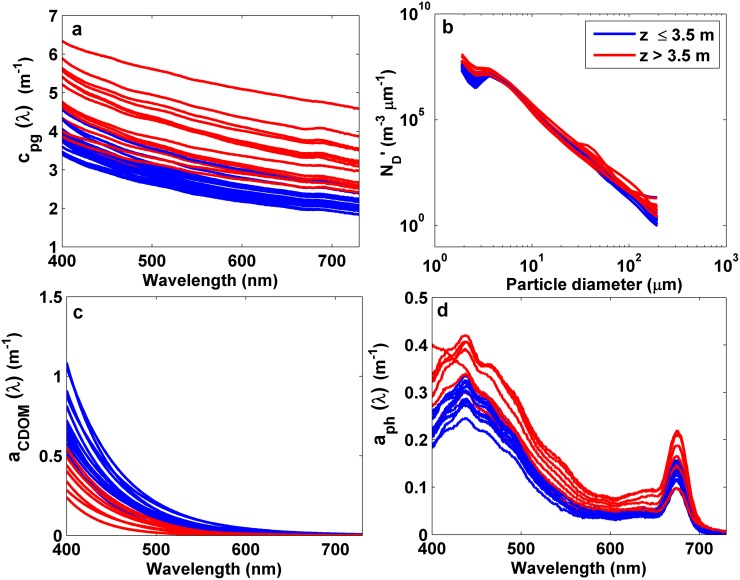
Optical properties measured at seven stations occupied in Alfacs Bay and over the water column. (a) In situ measured total non-water beam attenuation spectra. (b) LISST-derived size distribution for particle number. (c) CDOM absorption and (d) phytoplankton absorption spectra. Blue and red lines indicate measurements performed above and below the pycnocline, respectively.

The stratification of the water column determined the spatial variations observed in the optical properties. Thereby, significant differences in the beam attenuation spectra as well as in CDOM and phytoplankton absorption spectra were found between surface and bottom water layers (i.e. z ≤ 3.5 m and >3.5 m, respectively) (p<0.01) (Fig 7). Meanwhile, no noticeable differences were detected in the non-algal particulate absorption, a_nap_(λ) (not shown). Waters below the pycnocline were characterized by a higher attenuation and phytoplankton absorption, whereas CDOM absorption was lower. The shape of the particle size distribution was relatively homogeneous, although the PSD slope decreased slightly with depth (Table 1) (p>0.05).

**Table 1:** Mean and standard deviation of the non-water beam attenuation-based proxies and biogeochemical variables for waters above and below the pycnocline (i.e. z ≤ 3.5 m and z > 3.5 m, respectively).

	Z ≤ 3.5 m		Z > 3.5 m	
	Mean	SD	Mean	SD
**c**_**pg**_ **(710) (m**^**-1**^**)**	2.12	0.16	3.53	0.55
**c**_**pg**_**(685) peak height (m**^**-1**^**)**	0.074	0.007	0.12	0.024
**c**_**pg**_ **slope**	1.04	0.08	0.76	0.12
**PSD slope**	3.89	0.09	3.77	0.17
**TSM (g·m**^**-3**^**)**	3.97	1.37	9.36	3.44
**Chl-a (mg·m**^**-3**^**)**	5.28	1.44	9.47	3.73
**a**_**CDOM**_ **(443) (m**^**-1**^**)**	0.38	0.09	0.19	0.08

**Fig 8:**
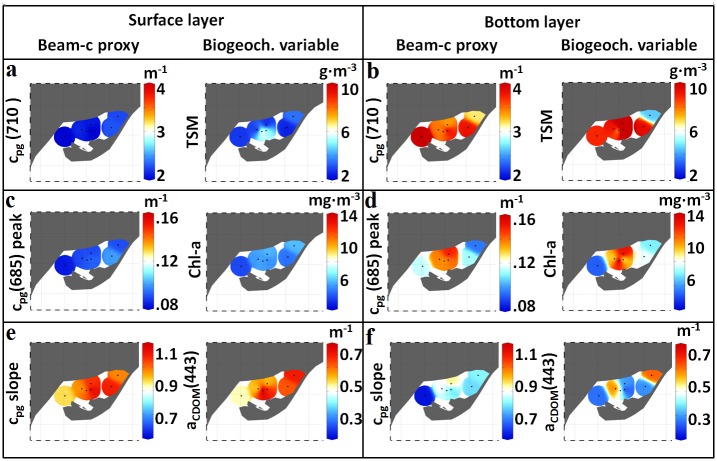
Spatial distribution of proxies from non-water beam attenuation and biogeochemical variables. Attenuation at 710 nm, c_pg_(710), *vs*. TSM at the (a) surface (z ≈ 0.5 m) and (b) bottom (z ≈ 5 m) layers of Alfacs Bay. c_pg_(685) peak *vs*. Chl-a concentration at the (c) surface and (d) bottom layers. c_pg_ spectral slope *vs*. CDOM absorption at 443 nm at the (e) surface and (f) bottom layers. Produced with Ocean Data View software [60].

**Fig 9:**
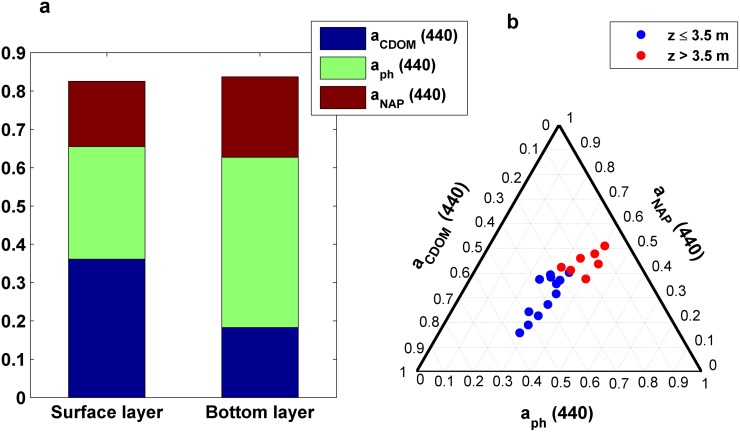
Analysis of partitioned absorption data. (a) Contribution of the major biogeochemical variables to the total non-water absorption coefficient at 440 nm for the two different water layers. (b) Ternary plot of the partitioned absorption coefficient at 440 nm (CDOM, phytoplankton, and non-algal particles) measured at the different sampling stations in Alfacs Bay. Blue and red circles correspond to water samples collected above and below the pycnocline, respectively.

The analysis of the spatial patterns based on c_pg_(λ) measurements was carried out by using the above-mentioned attenuation spectral features -c_pg_(710), c_pg_(685) peak and c_pg_ slope- and their relationships with the underlying biogeochemistry. In general, surface waters presented steeper c_pg_ slopes and lower values of both c_pg_(685) peak and c_pg_(710). In contrast, c_pg_(λ) measured below the pycnocline showed the opposite behavior, although their oscillations were larger (Table 1, Fig 8). Statistically significant differences (p<0.01) between surface and bottom layers were found in c_pg_(710) and c_pg_ slope, which increased by 40% and decreased by 27% with depth, respectively (Table 1, Fig 9a). The observed decrease in the attenuation spectral slope with depth can be associated not only with a reduction in CDOM contribution but also with resuspension events. Horizontally, except slight differences detected in the bottom layer, no significant spatial patterns were observed along the different bay regions, suggesting a horizontal homogeneity in c_pg_(λ) spectral features (Fig 8).

On the other hand, the spatial variability observed in the biogeochemical parameters over the sampled transects were in agreement with the total non-water beam attenuation proxies, since noticeable differences were found between surface and bottom water layers for these as well. While TSM and Chl-a increased with depth, CDOM absorption decreased (Table 1, Fig 8). However, horizontal variations observed in the attenuation proxies were less pronounced than those in the biogeochemical variables (Fig 8).

Finally, the results from the laboratory-measured absorption spectra exhibited differences in optical constituents contribution between surface and bottom layers, although the averaged magnitude of the total absorption was very similar for both cases (0.82 and 0.84 m^-1^, respectively) (Fig 9a). The spatial variations found in these variables coincided with those observed from the beam attenuation-based analysis. The ternary plot of the partitioned absorption at each sampling station showed that surface waters were characterized by a higher proportion of CDOM and lower phytoplankton absorption than the water below the pycnocline, which presented larger particulate fraction (Fig 9b). These observations are consistent with a previous study in the region [31], that found similar vertical distribution of optically active constituents (i.e. Chl-a, TSM, and CDOM).

### Temporal variability

**Fig 10:**
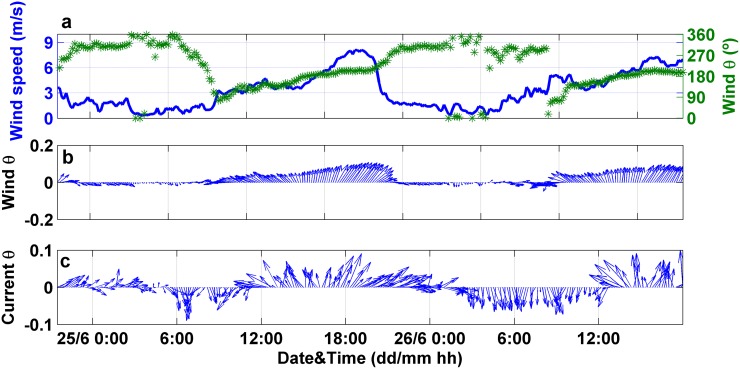
Time series of physical forcings along 48 hours. (a) Wind speed and direction measured in the weather station located in Alcanar. (b) Wind speed and direction (to) represented by arrows. Upward pointing arrow indicates the North. (c) Surface current velocity and direction represented by arrows, measured at 0.5 m depth with the ADCP located in the sampling station.

The 48 hours-time series of wind data showed a clear bimodal pattern with two dominant directions, from southwest and northwest (Fig 10a and 10b). NW winds blew for ca. 10–12 hours during the nighttime (from 10 pm until 9:30 am, approximately) and shifted from SE to SW during daytime. The velocities reached by southern winds were 5 m·s^-1^ on average, with maximum values up to 8 m·s^-1^ in the evening (8:30 pm). The strongest winds blew from SW from 5 pm to 10 pm. The observed wind pattern responded to the typical land breeze characterized by weak nocturnal winds (<2 m·s^-1^) blowing from land, that reverses the direction and increases the intensity during the daytime (sea breeze) [30, 33]. A similar behavior was observed in the surface current velocities, indicating that the water circulation at the sampling station was driven by the prevailing wind (Fig 10c). Thereby, surface water flowed in northward direction during daytime in response to southern winds and southwards when the wind ceased during nighttime. This pattern was observed within the first 2 m depth, though the velocity decreased with depth.

**Fig 11:**
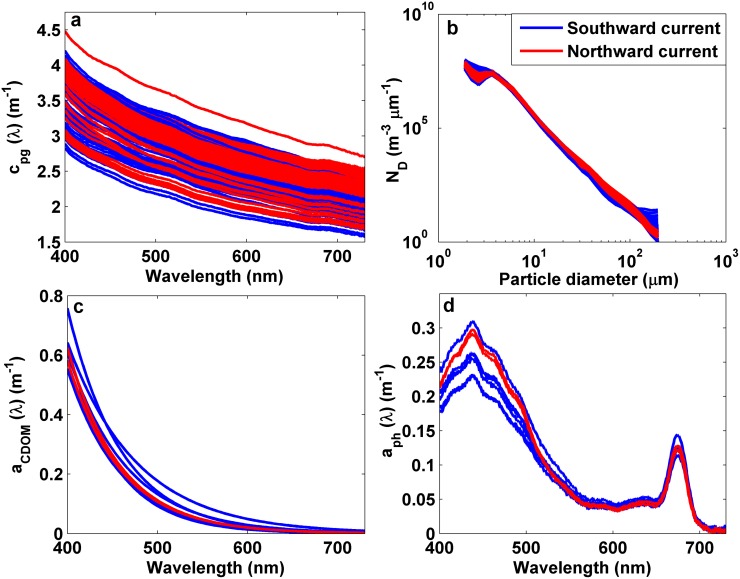
Optical measurements collected during profiling along 48 hours for the analysis of temporal patterns in Alfacs Bay. (a) In situ measured total non-water beam attenuation spectra. (b) LISST-derived size distribution for particle number. (c) CDOM absorption and (d) phytoplankton absorption spectra. Blue and red lines indicate measurements performed under the influence of southward and northward currents, respectively.

The effect of hydrodynamics on the water optical properties was analyzed for time and depth, and no significant correlations (p>0.05) between the optical properties and both the current velocity and direction were found. Thereby, the differences in the optical parameters between both prevailing flow regimes (i.e. southward and northward currents) were not statistically significant (p>0.05) (Fig 11).

**Fig 12:**
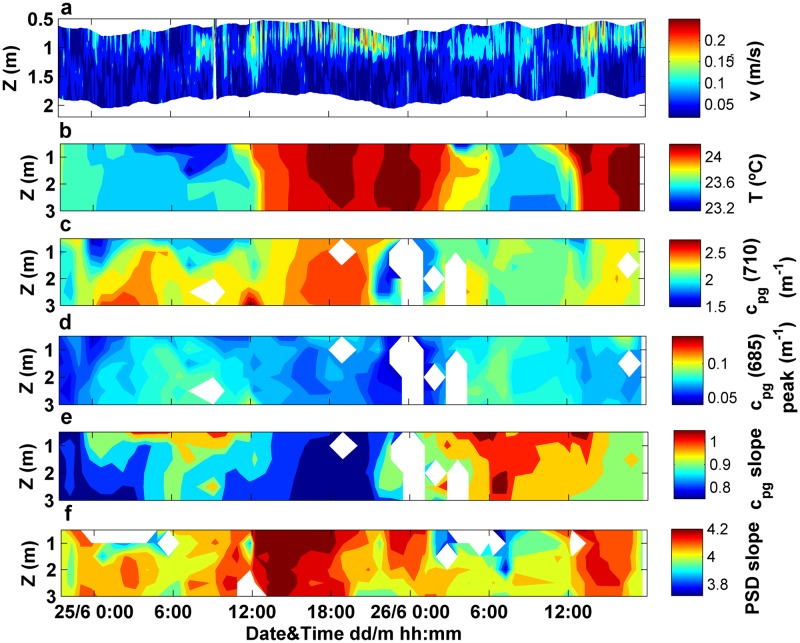
Variations with time and depth in the attenuation-based proxies and PSD slope along with the current velocity. (a) Temporal dataset of current velocity measured within the first 2 m depth. Time series of vertical profiles of (b) water temperature, (c) c_pg_(710), (d) c_pg_(685) peak, (e) c_pg_ spectral slope and (e) LISST-derived PSD slope.

The surface current velocity (at 0.5 m depth) was 0.1 m·s^-1^ on average, with a maximum value of 0.2 m·s^-1^, coinciding with SW winds episodes (Fig 12a). In relation to the hydrographical variability, water temperature showed a marked diurnal cycle (day-night), with an oscillation of 1.2°C. It ranged from 23.2°C (registered at 4 am) to 24.4°C, at 6 pm (Fig 12b). While no significant correlations were found between physical and optical variables, the time series of c_pg_(710) and particle size distribution slope were characterized by an increase in their magnitude at periods of maximum current velocities, in response to the more intense northward currents (Fig 12-a, 12-c and 12-f). In contrast, c_pg_(685) peak and c_pg_ spectral slope exhibited a bimodal pattern similar to that observed for the wind and current data. Both parameters rose under weak southward current conditions, whereas the minimum values concurred with the maximum current velocities flowing northwards (Fig 12-a, 12-d and 12-e).

**Fig 13:**
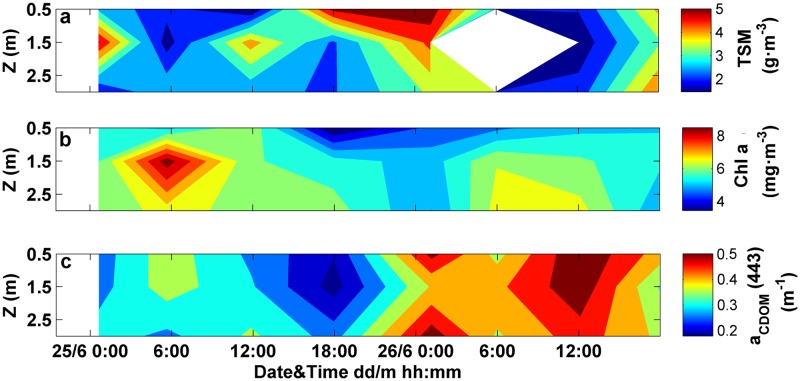
Variations with time and depth in biogeochemical variables. Time series of vertical profiles of (a) suspended matter concentration, (b) Chl-a concentration and (c) CDOM absorption at 443 nm.

The temporal variations in biogeochemical properties showed similar patterns as those observed based on c_pg_ proxies (Fig 13). The concentration of particulate matter increased with current velocity within the time interval from 6 pm to 12 am, on June 25, in agreement with c_pg_(710). Nevertheless, the magnitude of this increase differed, since c_pg_(710) showed a rise of 20% with respect to the mean value, whereas a 40% increase was detected for TSM concentration (Fig 13a). Apart from this, no similar patterns were observed between both parameters along the time series, involving no significant correlation between TSM and c_pg_(710) (p>0.05). In contrast, significant correlations were found between Chl-a concentration and c_pg_(685) peak (p<0.05), as well as between CDOM absorption at 443 nm and the attenuation spectral slope (p<0.01). The bimodal pattern detected in the optical proxies was also observed within the bulk analyses of Chl-a and a_CDOM_(443). Both variables increased their magnitude during southward current conditions (i.e. from 12 am to 12 pm, approximately) (Fig 13b and 13c).

## Conclusions

Continuous measurements of spectral beam attenuation coefficient collected in situ with an advanced-technology transmissometer have been proven as a powerful tool to better understand the existing interactions between physical and biogeochemical variables in the complex estuarine waters of Alfacs Bay (NW Mediterranean). In particular, this approach allowed the detection of qualitative changes in the major biogeochemical variables (i.e. Chl-a, TSM and CDOM) at high temporal and spatial scales in this microtidal estuary. Spatial patterns observed in the biogeochemical properties were driven by the vertical stratification of the water column. Accordingly, surface and bottom water layers were characterized by a different relative contribution of the major biogeochemical variables to the bulk beam attenuation. Meanwhile, observations along the 48 hours time series revealed a coupling between physical (meteorological and hydrodynamic conditions) and biogeochemical properties, since the prevailing hydrodynamic regimes determined the variations in water composition. The temporal and spatial patterns were obtained based on the spectral features of the total non-water beam attenuation coefficient and validated with laboratory results of discrete water samples (i.e. biogeochemical variables and partitioned absorption coefficients). Significant linear relationships were found between the non-water beam attenuation proxies and the biogeochemical variables. However, for future studies, it is highly recommended to include in situ beam attenuation measurements of 0.2 μm-filtered seawater for better CDOM characterization. The proposed proxies are subject to numerous uncertainties due to several factors affecting the attenuation signal (CDOM absorption and particle characteristics such as size, shape, composition, etc., which determine their absorption and scattering properties). For this reason, the collection of discrete water samples for laboratory analysis of biogeochemical variables is required for validation purposes.

Our results based on a high-frequency, low power (≤ 3 W), compact, versatile (adaptable to different observing platforms) and cost-effective (~10000€) beam attenuation meter, as well as on a simple and rapid data processing method, have demonstrated a capability to improve the operational monitoring of coastal waters towards a better understanding of their complex physical and biogeochemical interactions.
